# Sex determination based on morphometric measurements in yellow-legged gulls (*Larus michahellis*) around Istanbul

**DOI:** 10.1186/s40850-022-00133-w

**Published:** 2022-06-24

**Authors:** Gülsün Pazvant, Nazan Gezer İnce, Ermiş Özkan, Ozan Gündemir, Kozet Avanus, Tomasz Szara

**Affiliations:** 1 Department of Anatomy, Faculty of Veterinary Medicine, Istanbul University-Cerrahpasa, Istanbul, 34500 Turkey; 2 Department of Animal Breeding and Genetics, Faculty of Veterinary Medicine, Istanbul University-Cerrahpasa, Istanbul, 34500 Turkey; 3 Department of Morphological Sciences, Institute of Veterinary Medicine, Warsaw University of Life Sciences—SGGW, 02-776 Warsaw, Poland

**Keywords:** Avian anatomy, Discriminant functions, Yellow-legged gull, *Larus michahellis*, Sexual dimorphism, Veterinary anatomy

## Abstract

**Background:**

Yellow-legged gulls (*Larus michahellis*), commonly found in Istanbul and their surroundings, have a monomorphic plumage, like other gull species. For this reason, sex determination cannot be made externally. In this study, a total of 60 adult Yellow-legged gulls, 33 males, and 27 females, collected from the coastal areas of Istanbul, were examined. Discriminating functions were developed to classify males and females using birds that were previously sexed by DNA analysis and abdominal dissection.

**Results:**

Head length and bill depth were selected to build the discriminant function by the stepwise analysis. The function classified male gulls with an accuracy of 97.0% and females with an accuracy of 92.6%. Head length alone is the most accurate predictor in terms of the percentage of correct sex determination (90.9% for males, 92.6% for females).

**Conclusions:**

Functions that can easily determine sexual dimorphism for the population of *Larus michahellis* gulls around Istanbul have been put forward for the first time.

## Background

The population sexual structure in birds is an important parameter to be considered in ecological and behavioral studies. However, sex determination is very difficult for field researchers, breeders, and animal owners, in monomorphic species that do not reflect female or male characteristics in their appearance. Being able to accurately determine sex in monomorphic birds is critical for investigating certain aspects of their behavior such as sex-based survival analyses [1] and mate selection. Sexual dimorphism in the Yellow-legged gull (*Larus michahellis*) is indistinct [2]. Therefore, apart from observing the mating behavior, it is not possible to determine the sex by external observation. Sexing in birds is possible with laparoscopy (a minor surgical procedure) [3, 4], quantitative analysis of hormones in blood samples, or DNA diagnostics [5–7]. However, it has been reported that sex determination by a laparoscopic method not only harms animals but can even lead to their deaths [8]. Moreover, these methods are time-consuming, invasive, and require extensive laboratory equipment and specialist knowledge.

In monomorphic bird species, molecular diagnostic methods for sex determination are developed by examining various genes carried on sex chromosomes [9]. Birds have two sex chromosomes, W and Z. It is known that those who carry these chromosomes homozygously have male (ZZ) sexual characteristics, and those who carry heterozygously have female (ZW) sexual characteristics. In birds, the chromo helicase binding domain (CHD) genes on the W and Z chromosomes are conserved [9, 10]. Sex determination by molecular methods in non-ratite birds is based on the intron length differences of the CHD-W and CHD-Z genes [5, 6]. In recent years, studies based on sexual dimorphism in gulls have frequently used DNA diagnosis for the living and dissection for the dead ones [11–13].

Gulls have monomorphic plumage [2] and do not show pronounced sexual dimorphism, but there are dimensional differences between males and females [13, 14]. There are many studies in which an effective statistical evaluation (DFA – Discriminant Function Analysis) is used to decide which variables make the distinction between two groups by employing external morphology-based measurements to distinguish between sexes in gulls [12, 14–19]. The purpose of using discriminant function analysis (DFA) is to quantify the difference in body size and shape by combining morphometry from a set of external measurements that provide relatively accurate estimates of sex in birds.

In many morphometric studies, various measurements such as head length, bill depth, bill length, wing length, and tarsometatarsal length have been used for sex identification in gulls [11, 14, 15, 17, 18, 20, 21]. The first three measurements were stated in these studies to be the most useful in terms of DFA [16, 22–25]. However, it has been found that the various dimensional measurements differ geographically across many species [26–30]. Separate discriminant functions are needed for each species, subspecies or even population [13, 14, 19, 31, 32].

This study aims to determine the dimensional differences between male and female adult *Larus michahellis* gulls located around Istanbul by taking external body measurements. Specifically to:

1. determine the dimensional sexual dimorphism in the *Larus michahellis* gull population around Istanbul
2. present a reliable and easy-to-apply method to be used in field studies to determine the sex of adult Yellow-legged gulls.

## Results

Considering all morphometric measurements, it was determined that male gulls were larger and heavier than females. In addition, the greatest coefficient of variation (%CV) was observed in body weight (13.8% for males, 11.6% for females) and the smallest variation was observed in head length (2.7% for males and females) (Table 1).

**Table 1:** Measurements (weight—grams, linear measurements—mm) in male and female *Larus michahellis* gulls

	**Male**			**Female**			**P**	**%D**
	**n**	**Mean ± SD**	**%CV**	**n**	**Mean ± SD**	**%CV**		
Weight	33	771.55 ± 106.27	13.8	27	626.00 ± 72.69	11.6	< 0.001	18.9
WL	26	659.81 ± 41.24	6.3	23	636.09 ± 22.00	3.5	< 0.05	3.6
HL	33	126.67 ± 3.44	2.7	26	116.43 ± 3.17	2.7	< 0.001	8.1
BL1	33	80.17 ± 2.99	3.7	26	73.58 ± 2.93	4.0	< 0.001	8.2
BL2	33	63.46 ± 2.96	4.7	26	58.94 ± 2.02	3.4	< 0.001	7.1
BL3	33	56.63 ± 2.38	4.2	26	50.99 ± 2.04	4.0	< 0.001	9.5
N	33	22.12 ± 1.28	5.8	26	20.29 ± 1.08	5.3	< 0.001	8.3
BD1	33	18.81 ± 0.85	4.5	26	17.57 ± 0.84	4.8	< 0.001	6.6
BD2	33	19.77 ± 1.18	6.0	26	17.47 ± 0.87	5.0	< 0.001	11.6

Values that have statistical significance in discriminant function analysis are shown in Table 2. Head length was the most important factor for sex determination, correctly identifying 90.9% of males and 92.6% of females. Four functions were determined considering the accuracy of the original samples, including the morphometric measurements with the highest rates. These functions were created based on different measurements of head bill length commonly used in field studies.

**Table 2:** Accuracy of sex determination of Yellow-legged Gulls using measurements or discriminant functions

		**Accuracy of original samples**	
**Variables**	**Wilks’Lambda**	**Male**	**Female**
Weight	0.613	78.8% (26/33)	85.2% (23/27)
WL	0.760	81.8% (27/33)	70.4% (19/27)
HL	0.293	93.9% (31/33)	92.6% (25/27)
BL1	0.442	90.6% (30/33)	85.2% (23/27)
BL2	0.563	72.7% (24/33)	70.4% (19/27)
BL3	0.408	90.9% (30/33)	81.5% (22/27)
N	0.626	84.8% (28/33)	77.8% (21/27)
BD1	0.643	75.8% (25/33)	70.4% (19/27)
BD2	0.450	84.8% (28/33)	88.9% (24/27)
**Discriminant functions**			
D1 = HL*0.264 + BL1*0.061–36.999	0.289	93.9% (31/33)	92.6% (25/27)
D2 = HL*0.238 + BL3*0.142–36.702	0.278	93.9% (31/33)	92.6% (25/27)
D3 = HL*0.299 + BD1*0.012–36.744	0.293	93.9% (31/33)	92.6% (25/27)
D4 = HL*0.238 + BD2*0.419–36.924	0.253	97.0% (32/33)	92.6% (25/27)

The formulations created as a result of the DFA analysis are as follows:

$$\mathrm D1\:=\:\mathrm{HL}\ast0.264\:+\:\mathrm{BL}1\ast0.061\:-\:36.999$$

$$\mathrm D>0\;\mathrm{for}\;\mathrm{males},\;\mathrm D<0\;\mathrm{for}\;\mathrm{females}\;(\mathrm{centroid}\;1.370\;\mathrm{for}\;\mathrm{males},\;-1.739\;\mathrm{for}\;\mathrm{females})$$

**Fig. 1:**
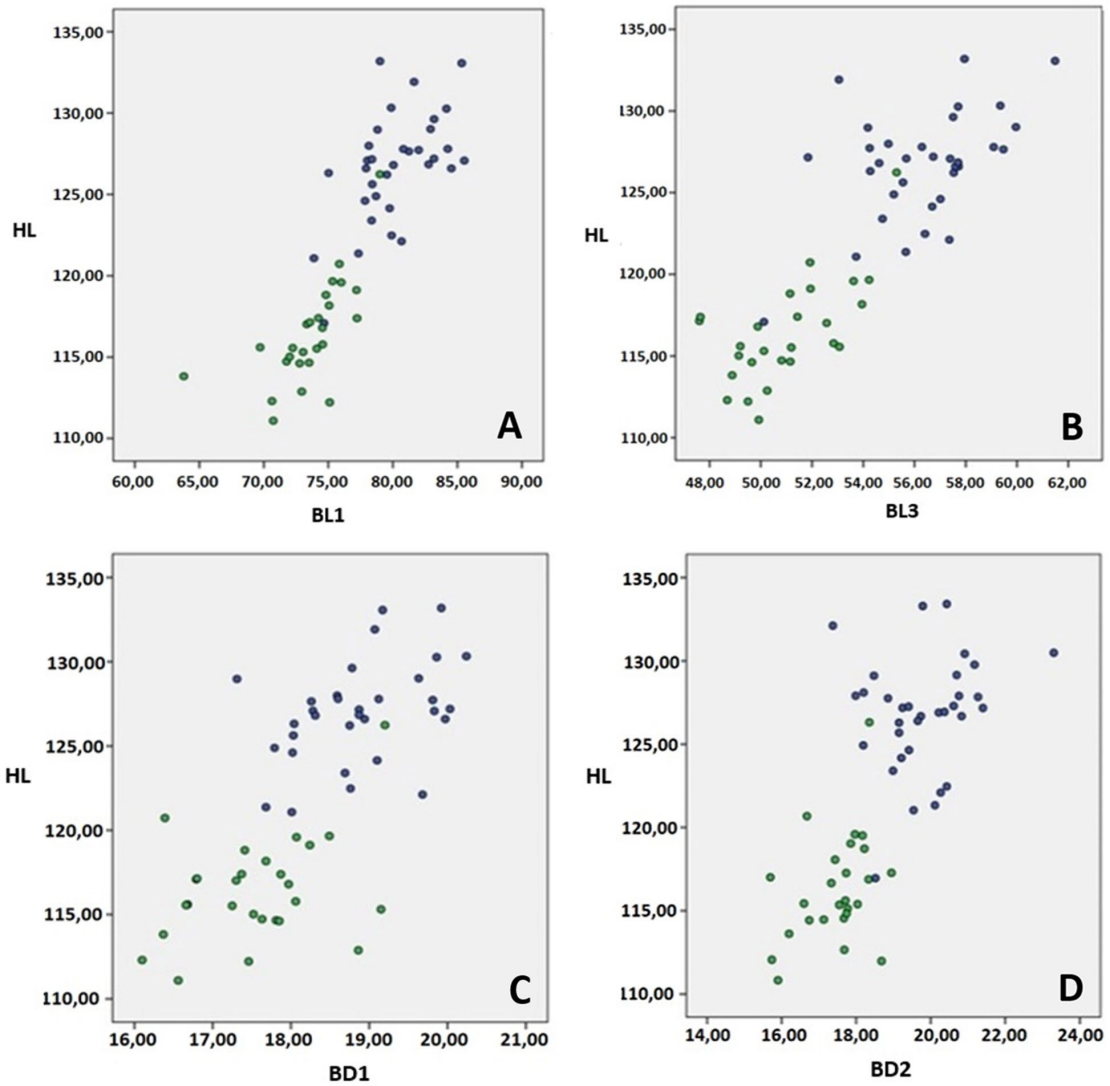
**A** The relationship between head length (HL) and bill length-1 (BL1) in function D1. **B** The relationship between head length (HL) and bill length-3 (BL3) in function D2. **C**. The relationship between head length (HL) and bill depth-1 (BD1) in function D3. **D** The relationship between head length (HL) and bill depth-2 (BD2) in function D4 (  : female;  : male)

The relationship between head length and bill length-1 (BL1) used in the D1 function is shown in Fig. 1A. In this analysis, 93.9% of males and 92.6% of females were correctly identified.

$$\mathrm D2\:=\:\mathrm{HL}\ast0.238\:+\:\mathrm{BL}3\ast0.142\:-\:36.702$$

$$\mathrm D>0\;\mathrm{for}\;\mathrm{males},\;\mathrm D<0\;\mathrm{for}\;\mathrm{females}\;(\mathrm{centroid}\;1.407\;\mathrm{for}\;\mathrm{male},\;-1.786\;\mathrm{for}\;\mathrm{female})$$

The relationship between head length and bill length-3 (BL3) used in the D2 function is shown in Fig. 1B. In this analysis, as in the D1 analysis, 93.9% of males and 92.6% of females were correctly identified.

$$\mathrm D3\:=\:\mathrm{HL}\ast0.299\:+\:\mathrm{BD}1\ast0.012\:-\:36.744$$

$$\mathrm D\:>\:0\;\mathrm{for}\;\mathrm{males},\;\mathrm D\:<\:0\;\mathrm{for}\;\mathrm{females}\;(\mathrm{centroid}\;1.356\;\mathrm{for}\;\mathrm{male},\;-1.721\;\mathrm{for}\;\mathrm{female})$$

The relationship between head length and bill depth-1 (BD1) used in the D3 function is shown in Fig. 1C. In this analysis, 93.9% of males and 92.6% of females were correctly detected.

$$\mathrm D4\:=\:\mathrm{HL}\ast0.238\:+\:\mathrm{BD}2\ast0.419\:-\:36.924$$

$$\mathrm D\:>\:0\;\mathrm{for}\;\mathrm{males},\;\mathrm D\:<\:0\;\mathrm{for}\;\mathrm{females}\;(\mathrm{centroid}\;1.498\;\mathrm{for}\;\mathrm{male},\;-1.902\;\mathrm{for}\;\mathrm{female})$$

The relationship between head length and bill depth-2 (BD2) used in the D4 function is shown in Fig. 1D. When the stepwise analysis was applied, the highest accuracy rate (97.0% for males and 92.6% for females) was observed in the D4 function (Table 2).

## Discussion

As described in the gull populations of the *Larus michahellis* species living in various geographical regions [13, 17, 33], all measurements of the same species in male gulls living around Istanbul were larger than in females. Male *Larus marinus* and *Larus crassirostris* may tend to be larger as a result of their more agonistic behavior, especially during nesting periods and being more aggressive throughout the season [34, 35].

In a study conducted on Baltic Herring Gull (*Larus argentatus argentatus*) in various age groups, it was stated that the most predictive sex features were head length and bill depth [11]. These two structures were also determined as the most defining characteristics of the sex in the Yellow-legged Gulls of the Istanbul region. However, in the use of formulations obtained in field studies, the rate of increase in bill depth and the fact that this increase continues until at least nine years of age, as in Herring Gulls [27], should be taken into consideration.

In many studies on morphological measurements of gull species, sex identification was desired, and various measurements such as total head length, bill depth, bill length, wing length, and tarsometatarsal length were used. The first three of these were stated to be the most useful measurement in terms of DFA for the studied gull populations [16, 22–25]. Bosch [17] stated that head length as a single measurement as well as combined in a function was the most predictive variable in sexual dimorphism in the Yellow-legged gull from the Mediterranean region. In our study, we built four discriminant functions that included head length, bill lengths, and depths that are relatively easy to record in the field. It was observed that head length was the only measurement that did not change in these formulations. Head length is therefore one of the most effective measurements in sex identification (90.9% in males, 92.6% in females).

Stepwise DFA provides high classification accuracy for *Larus michahellis* gulls using 2 measurements: head length and bill depth-2. However, their usefulness may be geographically limited. Because DFA analysis is obtained from a gull population, its application to species in different populations may be risky due to morphological differences between gulls of different origins. In short, the determination of which function is appropriate in sexual dimorphism varies depending on the population studied and the age structure of the population. Therefore, we suggest using the functions obtained in this study only for adult Yellow-legged gulls in and around Istanbul. It would be appropriate to test it first for use in *Larus michahellis* gull populations in wider contiguous areas.

Meissner et al. [11] determined that the wing length (WL) was the least dimorphic among the features that differed between the sexes and our results are similar (3.6%). This situation shows parallelism with the information obtained from studies on various gull species [31, 32, 37]. With the wear of the longest primary feather on the wing, wing length may vary seasonally [36, 37]. For this reason, the use of these data in field studies will not result in a reliable outcome after not being in the same season. Therefore, the use of age-related biometric measurements such as bill depth (i.e. depth of gonys) and wing length in DFA analysis requires attention.

The equations derived for adult gulls may fail to determine the sex of young individuals. As a result of this, it becomes clear that DFA based on morphological measurements should be applied not only in adults but also in various age groups to monitor the change depending on the time dimension. In addition, various external measurements vary geographically in many species [26–30]. In this study, it was observed that some linear measurements (bill length-1 and bill length-3) were not retained in stepwise DFA analysis, despite their ability to accurately predict sex, because the inclusion of these data in the function did not increase the accuracy rate. The absence of these measurements can be explained by the fact that the head length, which is closely related to the bill length, is already included in the discriminant function.

## Conclusions

With the data obtained in this study, functions that can easily determine sexual dimorphism for *Larus michahellis* gulls around Istanbul have been put forward for the first time.

## Materials and methods

### Study area

**Fig. 2:**
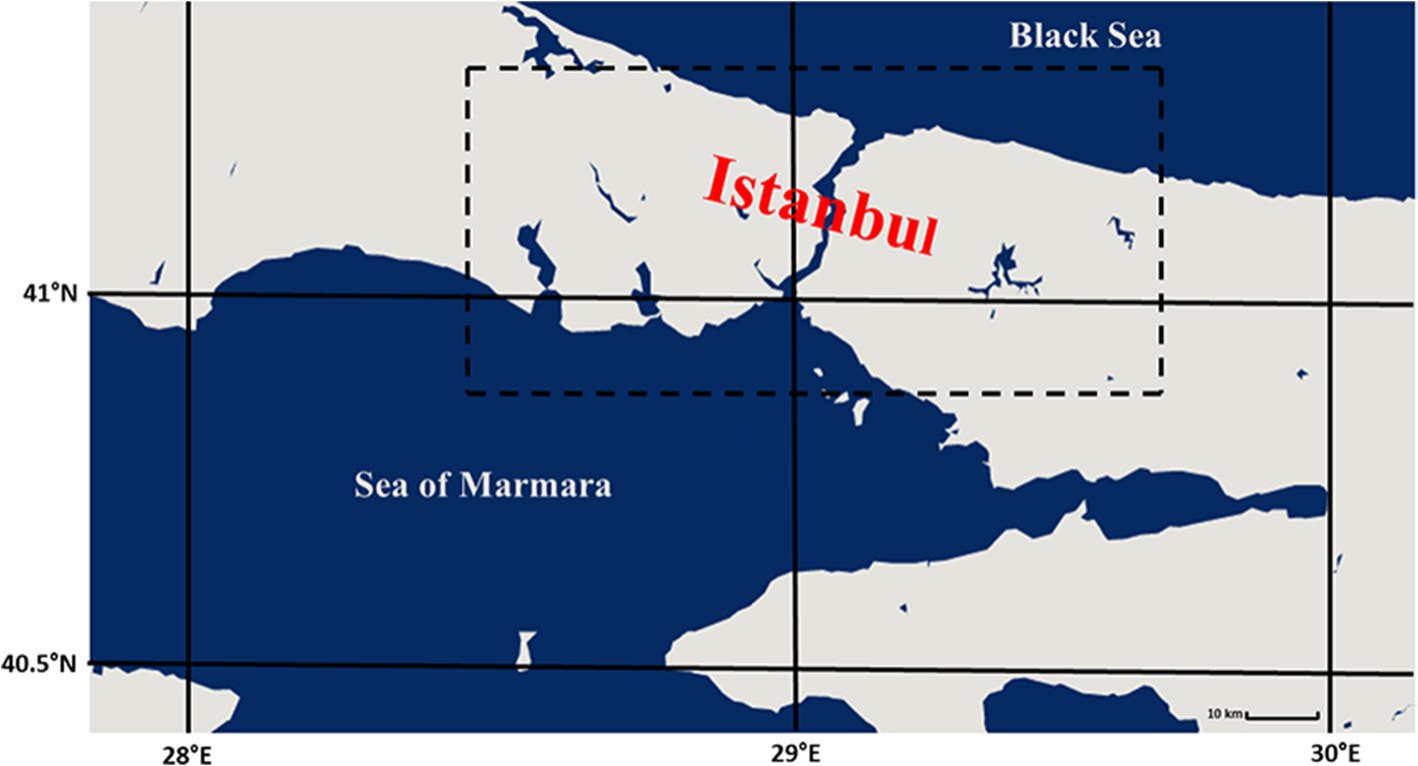
The area where the gulls were obtained

In this study, a total of 60 adult Yellow-legged gulls (Larus michahellis), 33 males, and 27 females were collected from the coastal areas of Istanbul (Fig. 2).

### Sex determination

Morphometric measurements were taken from the gulls whose sex was previously determined by DNA isolation of the live (5 females, 2 males) birds and abdominal dissection of the dead (22 females, 31 males) ones.

Genomic DNAs of animals were obtained from blood samples to perform sex determination in live gulls and to be used in molecular analysis. For this purpose, approximately 2 ml of blood samples were collected from the animals into sterile EDTA tubes from the medial metatarsal vein. Sex determination was performed by 2550F/2718R primer pairs [6] followed by imaging of the PCR products on 3% agarose gel.

### Morphometry

After the gulls were weighed on a digital scale to the nearest gram, morphometric measurements were taken from the head and wing with a digital caliper. Measurements were made by one researcher to minimize the measurement error. The variability in the number of N values depends whether a given structure was measured.

**Fig. 3:**
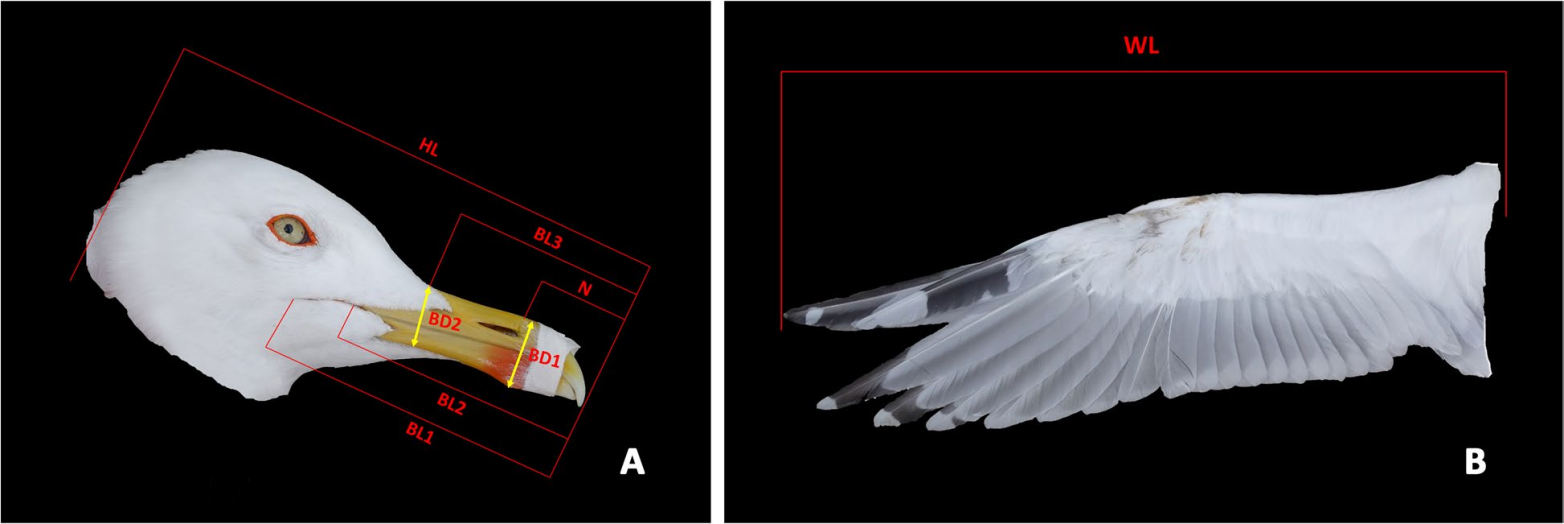
Morphometric measurements taken from the Yellow-legged Gull; **A**: HL (Head length); BL-1 (Bill length-1); N (Nalopsia); BL-2 (Bill length-2); BL-3 (Bill length-3); BD-1 (Bill depth-1); BD-2 (Bill depth-2); **B**: WL (Wing length)

The selection of measurements was made following previous work on sexing of gulls based on morphometric measurements [12, 14, 17, 21, 36]:

Head length (HL): The distance from the tip of the bill to the posterior end of the occipital prominence; Bill length-1 (BL-1): From the tip of the bill to the corner of the open mouth; Nalopsia (N): The distance from the tip of the bill to the nostril; Bill length-2 (BL-2): The length of the commissure from the tip of the bill to the feathering when the bill is closed; Bill length-3 (BL-3): The length of the culmen; Bill depth-1 (BD-1): The height of bill at gonys level; Bill depth-2 (BD-2): The height of the bill at the base of rhinotheca level; Wing length (WL): The distance between the base of the wing and the tip feather (Fig. 3).

### Data analysis

All statistical analyses were performed using IBM SPSS Statistics software version 28. The mean values of all morphometric data in male and female gulls were calculated. Then statistical comparisons between males and females were made using the unpaired Student's t-test. The percentage of dimorphism between the sexes for each measurement was calculated as:

%D = (Xm-Xf) / Xm.

Xm and Xf in this formula represent the mean values in males and females, respectively. The formulations using the morphometric measurements with the highest sexual dimorphism dimension were determined by linear DFA.A forward stepwise procedure based on minimization of Wilk’s lambda was used in the linear DFA to selectset of morphometric measurements which ensures most accurate differentiation between males and females. The criteria for entry and removal of the variable in the stepwise procedure were a *p*-value of 0.05 and 0.1 respectively. As a result. Individuals with D > 0 were classified as males and those with D < 0 were classified as females.

## Data Availability

Datasets are available from the corresponding author upon reasonable request.

## References

[1] Lens L, Galbusera P, Brooks T, Waiyaki E, Schenck T (1998). Highly skewed sex ratios in the critically endangered Taita thrush as revealed by CHD genes. Biodivers Conserv.

[2] del Hoyo J, Elliott A, Sargatal J (1992). Handbook of the birds of the world.

[3] Bush M, Fowler ME (1986). Laparoscopy and surgery. Zoo and Wild Animal Medicine.

[4] Richter NA, Bourne GR, Diebold EN (1991). Sex determination by body weight and linear measurements in American and Chilean Flamingos, previously surgically sexed: within-sex comparison to Greater Flamingo measurements. Zoo Biol.

[5] Griffiths R, Double MC, Orr K, Dawson RJG (1998). A DNA test to sex most birds. Mol Ecol.

[6] Fridolfsson AK, Ellegren H (1999). A simple and universal method for molecular sexing of non-ratite birds. J Avian Biol.

[7] Tomasulo AM, Del Lama SN, Rocha CD (2002). Molecular method of sexing waterbirds without DNA extraction. Waterbirds.

[8] Swengel SR, Ellis DH, Gee GF, Mirande CM (1996). Special techniques, C: Sex determination. Cranes: Their Biology, Husbandry, and Conservation.

[9] Griffiths R, Tiwari B (1993). The isolation of molecular genetic markers for the identification of sex. PNAS.

[10] Griffiths R, Korn RM (1997). A CHD1 gene is Z chromosome-linked in the chicken Gallus domesticus. Gene.

[11] Meissner W, Kośmicki A, Niemczyk A, Fischer I (2017). Development of sexual dimorphism and sexing of baltic herring gull (Larus argentatus argentatus) in successive age classes. Waterbirds.

[12] Galarza A, Hidalgo J, Ocio G, Rodriguez P (2008). Sexual size dimorphism and determination of sex in Atlantic yellow-legged gulls Larus michahellis lusitanius from northern Spain. Ardeola.

[13] Evans DR, Cavanagh PM, French TW, Blodget BG (1995). Identifying the sex of Massachusetts herring gulls by linear measurements. J Field Ornithol.

[14] Evans DR, Hoopes EM, Griffin CR (1993). Discriminating the sex of Laughing Gulls by linear measurements. J Field Ornithol.

[15] Fox GA, Cooper CR, Ryder JP (1981). Predicting the sex of Herring Gulls by using external measurements. J Field Ornithol.

[16] Coulson JC, Butterfield JE, Duncan N, Thomas N, Monaghan P, Shedden C (1983). The use of head and bill length to sex live gulls Laridae. Ibis.

[17] Bosch M (1996). Sexual size dimorphism and determination of sex in yellow-legged gulls. J Field Ornithol.

[18] Arizaga J, Aldalur A, Herrero A, Galicia D (2008). Sex differentiation of yellow-legged gull (Larus michahellis lusitanius): the use of biometrics, bill morphometrics and wing tip coloration. Waterbirds.

[19] Aguirre JI, Arana P, Antonio MT (2009). Testing effectiveness of discriminant functions to sex different populations of Mediterranean Yellow-legged Gulls Larus michahellis michahellis. Ardeola.

[20] Hallgrimsson GT, Helgason HH, Palsdottir ES, Palsson S (2016). Sexing adult and fledgling Lesser Black-backed Gulls from morphometrics. Ringing Migr.

[21] Gündemir O, Pazvant G, Gezer İN (2020). The morphometric examination of head area of black headed gulls (Larus Ridibundus) from Marmara Region. J Res Vet Med.

[22] Nugent G (1982). Sexing black-backed gulls from external measurements. Notornis.

[23] Hanners LA, Patton SR (1985). Sexing laughing gulls using external measurements and discriminant analysis. J Field Ornithol.

[24] Mawhinney K, Diamond T (1999). Sex determination of great black-backed gulls using morphometric characters. J Field Ornithol.

[25] Chochi M, Niizuma Y, Takagi M (2002). Sexual differences in the external measurements of Black-tailed Gulls breeding on Rishiri Island. Japan Ornithol Sci.

[26] Threlfall W, Jewer DD (1978). Notes on the standard body measurements of two populations of Herring Gulls (Larus argentatus). Auk.

[27] Coulson JC, Duncan N, Thomas CS, Monaghan P (1981). An age-related difference in the bill depth of Herring gulls Larus argentatus. Ibis.

[28] Renner M, Davis LS (1999). Sexing little penguins (Eudyptula minor) from Cook Strait, New Zealand, using discriminant function analysis. Emu.

[29] Kinsky FC, Falla RA (1976). A subspecific revision of the Australasian blue penguin (Eudyptula minor) in the New Zealand area. Records of National of the Museum of New Zealand.

[30] Meredith AM, Sin FYT (1988). Morphometrical analysis of four populations of the little blue penguin. Eudyptula minor J Nat Hist.

[31] Palomares LE, Arroyo BE, Marchamalo J, Sainz JJ, Voslamber B (1997). Sex- and age-related biometric variation of black-headed gulls, Larus ridibundus in western European populations. Bird Study.

[32] Hammouda A, Selmi S (2013). Morphometric sexing of Mediterranean Yellow-legged Gulls Larus michahellis michahellis breeding in the Gulf of Gabès, southern Tunisia. Ostrich.

[33] Isanmann P (1973). Bıometrische untersuchungen an der gelbfüssigen Silbormowe Larus argentatus michahellis aus der Camargue. Vogelwarte.

[34] Butler RG, Janes-Butler S (1983). Sexual differences in the behavior of adult great black-backed gulls (Larus marinus) during the pre-and post-hatch periods. Auk.

[35] Kazama K, Niizuma Y, Sakamoto KQ, Watanuki Y (2011). Factors affecting individual variation in nest-defense intensity in colonially breeding black-tailed gulls (Larus crassirostris). Can J Zool.

[36] Meissner W (2007). Differences in primary molt and biometrics between adult and second-year black-headed gulls in Puck Bay (southern Baltic). Waterbirds.

[37] Coulson JC (2009). Sexing black-legged kittiwakes by measurement. Ringing Migr.

